# Proinflammatory state and metabolic dysregulation linking delayed feeding progression to extrauterine restricted head growth in extremely preterm infants

**DOI:** 10.1186/s12916-025-04525-w

**Published:** 2025-12-29

**Authors:** Chih-Chia Chen, Yung-Chieh Lin, Cheng-Yang Lee, Cheng-Chin Kuo, Tzu-Hao Chang, Chao-Ching Huang

**Affiliations:** 1 Graduate Institute of Clinical Medicine, College of Medicine, National Cheng-Kung University, Tainan, Taiwan; 2 Department of Pediatrics, College of Medicine, National Cheng Kung University Hospital, National Cheng Kung University, Tainan, Taiwan; 3 Graduate Institute of Biomedical Informatics, Taipei Medical University, Taipei, 110 Taiwan; 4 Institute of Cellular and System Medicine, National Health Research Institutes, Zhunan, Taiwan; 5 Department of Bioscience Technology, Chung Yuan Christian University, Taoyuan, Taiwan; 6 Clinical Big Data Research Center, Taipei Medical University Hospital, Taipei, Taiwan; 7 Department of Pediatrics, College of Medicine, Shuang Ho Hospital, Taipei Medical University, Taipei, 23561 Taiwan

**Keywords:** Preterm infants, Feeding progression, Extrauterine growth restriction, Head size, MRI, Proinflammatory, Metabolomics

## Abstract

**Background:**

Growth of head circumference is critically associated with neurodevelopmental outcomes. Extrauterine growth restriction of head circumference from birth to term-equivalent age is linked to impaired neurodevelopment. This study examined whether a proinflammatory state and metabolic dysregulation characterize the association between delayed feeding progression and extrauterine restricted head growth in extremely preterm infants.

**Methods:**

This cohort study included infants born ≤ 28 weeks’ gestation between 2019 and 2021. Feeding progression trajectories, categorized as improvement or delayed improvement based on daily enteral feeding milk volumes during the first 8 weeks, were analyzed using kmlShape. Plasma metabolomics were assessed at 36 weeks postmenstrual age, and head growth and brain MRI were evaluated at term-equivalent age.

**Results:**

Among the 98 extremely preterm infants, 62 (63%) demonstrated improvement in feeding progression, while 36 (37%) had delayed improvement. Compared to the feeding improvement group, the delayed feeding improvement group had higher rates of gastrointestinal morbidities, including necrotizing enterocolitis (NEC) of Bell stage II or higher (17% vs. 2%, *p* = 0.009) and abdominal surgery for non-NEC events (25% vs. 8%, *p* = 0.021) during admission, and a significantly increased risk of extrauterine growth restriction in head circumference by term-equivalent age (47% vs. 23%, *p* = 0.021). The multivariable analysis showed delayed feeding improvement was also a significant risk associated with the delta *z*-scores below − 1.5 in head circumference (adjusted odds ratio [aOR]: 5.26 [95% CI 1.66–16.65]). MRI examinations revealed significantly smaller residual brain volumes involving total brain tissue volume, brainstem, and cerebellum in the delayed improvement group. Untargeted plasma metabolomics showed elevated levels of hydroxyeicosatetraenoic acid, leukotriene B4, prostaglandins, bile acids and immune markers, and reduced levels of L-tyrosine, phenylpyruvic acid, L-tryptophan metabolism, and L-carnitine biosynthesis were found in the delayed improvement group compared to that in the improvement group.

**Conclusions:**

Proinflammatory and dysregulated metabolic state following early delayed feeding progression were associated with impaired extrauterine head growth, highlighting the potential role of the immature gut-brain axis in preterm infants.

**Supplementary Information:**

The online version contains supplementary material available at 10.1186/s12916-025-04525-w.

## Background

Extremely preterm infants born at or before 28 weeks of gestation represent a highly vulnerable population. While their overall survival rate has improved in the recent decade, a significant number of them still suffer from growth and neurodevelopmental impairment at follow-up [1–3]. These infants are born during a critical period of fetal development in the third-trimester window, leaving their immature organs, such as the brain, heart, lung, intestine, and kidney, prone to adverse exposures in the neonatal intensive care unit (NICU) [4–8].

Although growth of body weight (BW) has been commonly used as a measure of nutrition, growth of head circumference (HC) is more closely related to neurodevelopmental outcomes due to its relevance to brain volume and cortical maturation [9–12]. Extrauterine growth restriction (EUGR) in head circumference (HC) has been linked to adverse neurodevelopmental outcomes at follow-up [12–17]. Increasingly, longitudinal definitions of EUGR—based on a decline in HC *z*-score of − 1, − 1.2, − 1.5, or − 2 standard deviations from birth to discharge or term-equivalent age (TEA), depending on severity—are recognized as more reliable predictors of neurodevelopmental impairment than static, percentile-based criteria [18–20]. Therefore, adequate nutritional support for growth during the period in the NICU is critical for extremely preterm infants [21–23]. Although nutritional gain by feeding advancement is sometimes affected by the clinician’s decision for a particular infant, nutritional practice guidelines for preterm infants following the recommendations from the European Society of Pediatric Gastroenterology, Hepatology and Nutrition (ESPGHAN) has been enforced in our hospital since 2015 [24]. The introduction and advancement of enteral feeding in extremely preterm infants is often delayed or interrupted because of prematurity-related risks, adverse exposures, and gastrointestinal (GI) morbidities [25–27]. Risks and exposures, such as sepsis, mechanical ventilation [28–31], and the functional immaturity of GI tracts may have significant impacts on feeding progression. In addition, GI morbidities, such as necrotizing enterocolitis (NEC) and non-NEC morbidities, may also change the enteral feeding trajectory differently [32]. Given that delayed feeding progression is a shared clinical manifestation resulting from this complex interplay of both gastrointestinal and systemic morbidities, it can be conceptualized as a surrogate marker. This marker can be used to early identify a group of preterm infants who are more vulnerable and physiologically unstable during their NICU stay.

Monitoring the patterns of enteral feeding progression and the related anthropometric change in the NICU is one of the important issues to improve growth and neurodevelopment outcomes of extremely preterm infants [23, 33–35]. Our previous studies demonstrated that early-life delayed improvement in feeding progression was linked to neurodevelopmental impairment in preterm infants [23, 34]. Whether delayed feeding progression has a selective impact on head growth remains unknown. If so, whether there are bioactive mediators involved in EUGR-HC after early-life delayed feeding improvement remains unclear [36, 37].

Mass spectrometry-based untargeted metabolomics has been used to obtain comprehensive metabolite profiling and metabolic pathways. By comparing the metabolite profiles of two or more disease phenotypes, metabolomics may identify the biomarkers related to the perturbation of specific diseases [38, 39]. Metabolomics has been applied for biomarker discovery in neonatal hypoxic brain injury in term-birth neonates and preterm birth recently [40–44]. One untargeted metabolomic study found alterations in plasma levels of glycerophospholipids, sphingolipids, and other lipid levels after EUGR in BW of preterm infants, and the biochemical species decreased progressively as the level of EUGR increased in severity [44]. However, very few studies have focused on alterations of proinflammatory mediators and metabolic pathways by plasma metabolomics in preterm infants who have EUGR in HC after delayed feeding progression [44]. Metabolic phenotyping by metabolomics could pave the way for developing targeted nutritional interventions for infants experiencing adverse feeding progression and inadequate HC growth in the NICU [45].

The impact of the immature gut-brain axis in preterm infants has received increasing recognition [46]. Using early feeding progression patterns and growth outcomes in BW and HC at TEA as a framework, this study tested the hypothesis that EUGR predominantly affects head growth, and that proinflammatory conditions, along with disrupted metabolic pathways, contribute to EUGR in HC following delayed improvements in feeding among extremely preterm infants. Additionally, the vulnerable brain regions affected by EUGR in HC were also assessed using brain MRI.

## Methods

### Study population

We conduct this prospective cohort study on extremely preterm infants who were born less than 29 weeks’ of gestation and admitted to the university hospital between 2019 and 2021. Infants with congenital abnormalities or genetic syndromes were excluded from analysis. Informed consents were obtained from the parents, and the institutional review board of National Cheng-Kung University Hospital approved this study (A-BR-108–013).

### Neonatal risks and morbidities during admission

Medical records were reviewed for demographics, neonatal risks, and morbidities. The demographics and neonatal risks included maternal age, antenatal steroid use, preeclampsia, small for the gestational age (SGA), clinical chorioamnionitis, gestational age, gender, birth anthropometry, multiple births, and Apgar scores. Neonatal morbidity encompassed severe brain injury, hemodynamically significant patent ductus arteriosus (hsPDA) requiring medical or surgical intervention, sepsis, NEC of Bell stage II or higher, non-NEC events requiring surgery (including volvulus, meconium ileus, spontaneous intestinal perforation, gastrointestinal bleeding, esophageal rupture, and congenital bands related intestinal herniation), bronchopulmonary dysplasia (BPD), and severe retinopathy of prematurity (ROP), defined as stage 2 plus or worse, or requiring retinal therapy (Additional File 1:Table S1) [34, 47–55].

### Enteral milk feeding protocol

A standard nutritional protocol that included parenteral nutrition and enteral feeding had been established for preterm infants in the NICU [34, 56]. In our hospital, we followed the ESPGHAN guideline since 2015 [24]. Enteral feeding began with trophic feeding, prioritizing the mother’s own milk, with donor human milk used when unavailable. Once the infant met the eligibility criteria and after evaluating the infant’s clinical condition, the daily enteral feeding volumes were gradually increased by 10–25 mL/kg/day. Advancement was paused or slowed based on clinical signs of feeding intolerance, such as bile-stained or increased gastric residuals, or abdominal distension. When the enteral feeding volume reached 100 mL/kg/day, human milk was fortified with a powdered human milk fortifier to a caloric density of approximately 0.8 kcal/mL, and intravenous total parenteral nutrition (TPN) was discontinued [34]. Intravenous fluids were stopped once enteral feeding reached the full feeding criteria (120 mL/kg/day). We documented the daily feeding amounts in the first 56 days after birth and converted the raw data to mL/kg/day by adjusting for daily BW. These longitudinal data were subsequently used for clustering analysis to classify infants into distinct feeding progression groups.

### Growth outcome measurement in body weight and head circumference by TEA

BW and HC *z-*scores are established using the population-based norm for HC at birth‍, a change in the *z*-score represents growth relative to the healthy reference fetus [57]. For preterm infants who are receiving critical care in the NICU, changes in growth using *z-*scores might reflect the growth outcomes of the individual preterm infant. The corresponding *z*-scores of BW and HC at birth and at TEA were evaluated using Fenton’s postnatal growth charts [47]. Delta *z*-scores were computed by subtracting the *z*-scores at birth from those at TEA. EUGR in BW and HC was defined, respectively, as delta *z*-scores below − 1.5 [23].

### Brain volume measurements by MRI

Brain MRI was assessed at TEA. Total brain volume encompassed all parenchymal brain regions. The cerebrospinal fluid comprised the lateral ventricle and other spaces such as the third and fourth ventricles, and extra-axial fluid. Intracranial volume accounts for the combined total of brain and cerebrospinal fluid volumes. Brain volume measurements were adjusted for sex and postmenstrual age. Residual brain volume indicated the difference between the measured brain volume and the expected volume based on linear regression, as described previously [58]. A brain size smaller than expected resulted in a negative residual, whereas a size larger than expected produced a positive residual.

### Metabolomics analysis

Plasma samples for metabolomics were obtained from extremely preterm infants at postmenstrual age 36 weeks, and from term-birth neonates at 2–3 days after birth. The untargeted metabolomic workflow comprised sample preparation and extraction, followed by liquid chromatography-mass spectrometry (LC–MS) spectroscopy and data processing (Additional File 1: Fig S1). Metabolomic analysis was conducted using a quadrupole-time-of-flight (QToF) mass spectrometry system, operated in both positive and negative electrospray ionization modes with a resolution exceeding 10,000. LC–MS software, ProgenesisQI (Waters Corporation), was utilized to acquire mass spectrometry data. A comprehensive approach was implemented utilizing several advanced techniques. Partial Least Squares Discriminant Analysis (PLS-DA) was employed to identify patterns and classify the data based on metabolic profiles [59]. Volcano plots were utilized to visualize the statistical significance and fold changes of metabolites.

### Statistical analyses

#### Clinical data and feeding progression patterns

Continuous variables were reported as mean ± SD and analyzed using independent *t*-test or Mann–Whitney *U* test, depending on whether the normal distribution assumption was met. Categorical variables were presented as numbers (%) and compared using the *χ*^2^ test or Fisher’s exact test. The feeding trajectories were assessed by examining daily feeding volumes using the “kmlShape” package in R. The analyses allowed grouping individuals whose trajectories have similar forms but shifted positions in time [60] and had been applied to stratify the heterogenic trajectories within the study populations according to the shapes after examining their time-series and longitudinal data [34, 60, 61]. Based on the resulting patterns, these groups were labeled “improvement” and “delayed improvement” group. A repeated measures analysis using a linear mixed model was conducted to examine the effects of group and postnatal days on feeding volume. Logistic regression models were employed to calculate the odds ratios and 95% confidence intervals (CI) for the neonatal risks associated with delta *z*-scores of HC less than − 1.5.

#### Metabolomics

The intensity table of samples and metabolites was exported by ProgenesisQI software, and one factor statistical analysis was performed using MetaboAnalyst (https://www.metaboanalyst.ca/MetaboAnalyst/ModuleView.xhtml). The log2-fold change of the delayed improvement and improvement groups and the Wilcoxon test *p*-value were calculated. Finally, differential abundance metabolites (DAMs) were identified by fold change >  = 1.3 or <  =  − 1.3, and p-value <  = 0.05. Differentiation between the two feeding trajectory groups was performed by HMDB database mapping using ProgenesisQI, and compounds with predictive scores of at least 36 were retained. The predominance of partial least squares-discriminant analysis (PLS-DA) was used to analyze metabolomics datasets. These compounds (DAM-related compounds) were then converted from HMDB to KEGG IDs for pathway enrichment analysis using MetaCore™ (Clarivate). FDR was used in the metabolite functional enrichment analysis, and the difference in metabolites was calculated using the Wilcoxon rank-sum test in MetaboAnalystR [62]. We focused on pathways, including glycolysis, tricarboxylic cycle, pentose phosphate pathway, lipid and amino acid metabolism, to represent metabolite differences between the two different feeding progression groups.

## Results

### Neonatal risks and morbidities associated with delayed improvement in feeding progression

**Fig. 1:**
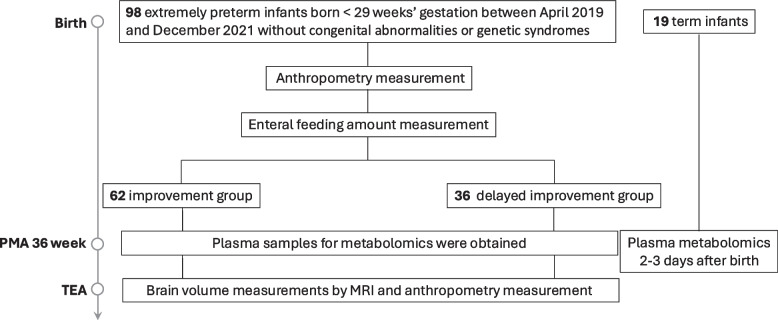
Flow diagram of the extremely preterm infant study: feeding, growth, and metabolomics. PMA, postmenstrual age; TEA, term equivalent age

**Fig. 2:**
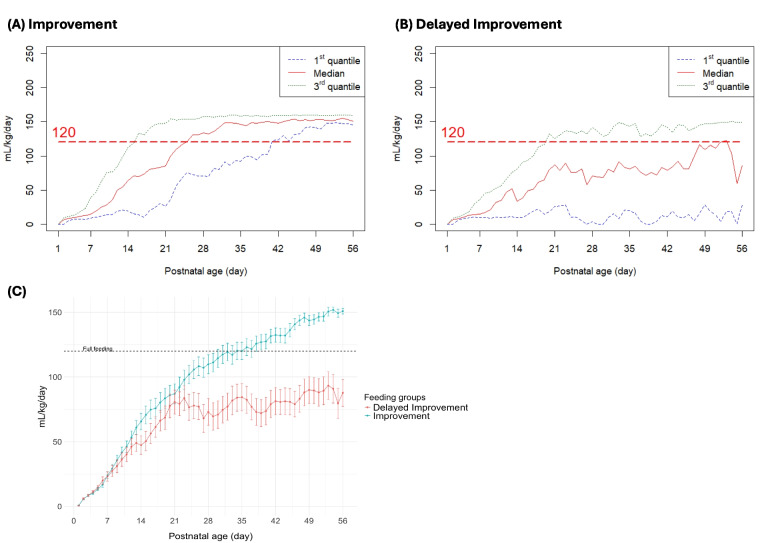
The enteral feeding progression trajectories. The trajectory of feeding progression was categorized as improvement (*n* = 62) (**A**), and delayed improvement (*n* = 36) (**B**) by clustering analysis of the daily enteral feeding volumes in the first 56 postnatal days, shown the median, 1st quantile (Q1) and 3rd quantile (Q3) for each day. Full feeding was defined as 120 mL/kg/days (the red line). (**C**) Distinct feeding progression patterns were observed between the improvement and delayed improvement groups, as demonstrated by a significant interaction between group and postnatal days on feeding volume (*p* < 0.001) using a linear mixed model

Of the 98 extremely preterm infants included for the analysis of feeding trajectory (Fig. 1), 62 infants had improvement (Fig. 2A), and 36 infants displayed delayed improvement in feeding progression (Fig. 2B). Repeated measures analysis using a linear mixed model demonstrated a significant interaction between group and postnatal days on feeding volume (*p* < 0.001), indicating different feeding progression patterns between the two groups (Fig. 2C). The median postnatal age reaching full feeding was much earlier in the improvement group compared to that in the delayed improvement group (24 days vs. 52 days). The improvement group also had a significantly higher percentage of infants reaching full feeding by 8 weeks of postnatal age (98% vs. 83%, *p* = 0.009) (Table 1).

**Table 1:** Demographics, neonatal risks, and morbidities during admission between the improvement and the delayed improvement group

**Variable**	**Feeding improvement** ***N* = 62**	**Delayed feeding improvement** ***N* = 36**	*p* -value
**Perinatal risks**			
Maternal age, mean ± SD	32.6 ± 5.9	33.4 ± 4.4	0.508
Preeclampsia,* n* (%)	11 (18)	5 (14)	0.619
Clinical chorioamnionitis, *n* (%)	13 (21)	8 (22)	0.884
Multiple gestation, *n* (%)	34 (55)	18 (50)	0.644
Antenatal steroid, *n* (%)	62 (100)	35 (97)	0.367
**At birth**			
Gestational age, weeks, mean ± SD	25.9 ± 1.9	25.8 ± 1.9	0.815
Body weight, gm, mean ± SD	849 ± 238	841 ± 236	0.885
Head circumference, cm, mean ± SD	23.5 ± 2.3	23.4 ± 2.1	0.767
Male, *n* (%)	34 (55)	22 (61)	0.545
Small for gestational age, *n* (%)	5 (8)	4 (11)	0.721
5-min Apgar score < 7, *n* (%)	18 (29)	15 (42)	0.202
**Morbidity during admission**			
hsPDA requiring ligation, *n* (%)	13 (21)	15 (42)	0.029
Severe brain injury, *n* (%)	7 (11)	6 (17)	0.540
Sepsis, *n* (%)	14 (23)	10 (28)	0.564
NEC (Bell stage ≥ II), *n* (%)	1 (2)	6 (17)	0.009
Non-NEC events requiring surgery, *n* (%) ^a^	5 (8)	9 (25)	0.021
**Feeding progression**			
Age at initial feeding, postnatal days, mean ± SD	3.0 ± 2.8	2.8 ± 2.3	0.792
% of infants reaching full feeding by 8 weeks of postnatal age, *n* (%)	61 (98)	30 (83)	0.009
**Postmenstrual age 36 weeks**			
Severe ROP, *n* (%)	10 (16)	8 (22)	0.453
Moderate/severe BPD, *n* (%)	42 (68)	31 (86)	0.044

*hsPDA*, hemodynamically significant patent ductus arteriosus; *Severe brain injury*, intraventricular hemorrhage grade III, any grade of intraventricular hemorrhage plus periventricular hemorrhage, or cystic periventricular leukomalacia; *NEC*, necrotizing enterocolitis; *ROP*, retinopathy of prematurity, *Severe ROP*, ROP stage ≥ II plus; *BPD*, bronchopulmonary dysplasia, BPD defined by *Jensen* 2019 definition, according to the types of respiratory support (grade 1, nasal cannula ≤ 2 L/min; grade 2, Nasal cannula > 2 L/min or nCPAP or nasal IPPV; and grade 3, invasive ventilator) assessed at 36 weeks’ PMA. Moderate/severe BPD: BPD grade ≥ II. Full feeding was defined as enteral feeding reaching 120 mL/kg/day. Student *t* test was used for continuous variables; chi-square test was used for categorical variables

^a^The non-NEC events requiring surgery included volvulus (*n* = 4), meconium ileus (*n* = 5), spontaneous intestinal perforation (*n* = 2), gastrointestinal bleeding (*n* = 1), esophageal rupture (*n* = 1), and congenital bands related intestinal herniation (*n* = 1)

The perinatal and neonatal risk factors were comparable between the two groups (Table 1). The delayed improvement group was more prone to have hsPDA requiring ligation (42% vs. 21%, *p* = 0.029), NEC of Bell stage II or higher (17% vs. 2%, *p* = 0.009), abdominal surgery for non-NEC events (25% vs. 8%, *p* = 0.021), and moderate to severe BPD (86% vs. 68%, *p* = 0.044) than the improvement group by 36 weeks of postmenstrual age.

### Differences in head growth by TEA between the two feeding progression groups

The mean *z*-scores of BW at birth and at TEA were similar between the two different feeding improvement groups (Table 2). Despite the two groups had comparable HC *z*-scores at birth, the delayed improvement group was significantly lower in the *z*-score (− 1.79 ± 1.36 vs. − 1.04 ± 1.26, *p* = 0.007) and delta *z*-score (− 1.54 ± 1.46 vs. − 0.78 ± 1.33, *p* = 0.011), and had a higher proportion of infants with delta *z*-scores <  − 1.5 (47% vs. 23%, *p* = 0.021) in HC at TEA than the improvement group. In the multivariable model, delayed improvement in feeding was also a significant risk associated with the delta *z*-scores below − 1.5 in HC (adjusted odds ratio [aOR]: 5.26 [95% CI 1.66–16.65]), after adjusting for gestational age, multiple gestation, and sepsis (Table 3).

**Table 2:** Differences in growth outcomes at term equivalent age between the improvement and delayed improvement groups

	**Feeding improvement**	**Delayed feeding improvement**	*p*-value
**At birth**			
Body weight, *z*-score, mean ± SD	− 0.17 ± 0.79	− 0.18 ± 0.74	0.966
Head circumference, *z*-score, mean ± SD	− 0.26 ± 0.98	− 0.31 ± 1.01	0.822
**At term equivalent age**			
Body weight			
*Z*-scores, mean ± SD	− 0.70 ± 1.25	− 1.08 ± 1.22	0.145
Delta *z*-scores, mean ± SD	− 0.53 ± 1.01	− 0.91 ± 1.02	0.080
Delta *z*-scores < − 1.5, *n* (%)	10 (16)	9 (25)	0.420
Head circumference			
*Z*-scores, mean ± SD	− 1.04 ± 1.26	− 1.79 ± 1.36	0.007
Delta *z*-scores, mean ± SD	− 0.78 ± 1.33	− 1.54 ± 1.46	0.011
Delta *z*-scores < − 1.5, *n* (%)	14 (23)	17 (47)	0.021

**Table 3:** The risks for delta *z*-score of head circumference <  − 1.5 between birth and term-equivalent age

Variable	OR [95% C.I.]	*p*-value	aOR [95% C.I.]	*p*-value
Gestational age	0.91 [0.87, 0.95]	< 0.001	0.65 [0.48, 0.87]	0.004
Male	0.92 [0.76, 1.12]	0.408	NA	0.255
Small for gestational age	0.93 [0.66, 1.30]	0.663	NA	0.189
Multiple gestation	1.27 [1.06, 1.52]	0.013	4.19 [1.28, 13.70]	0.018
NEC (Bell stage ≥ II)	1.31 [0.92, 1.88]	0.141	NA	0.659
Sepsis	1.59 [1.30, 1.93]	< 0.001	5.82 [1.81, 18.73]	0.003
Moderate/severe BPD	1.31 [1.06, 1.61]	0.013	NA	0.680
Severe brain injury	1.41 [1.08, 1.84]	0.014	NA	0.435
Delayed improvement in feeding progression	1.30 [1.07, 1.57]	0.008	5.26 [1.66, 16.65]	0.005

### Impact of delayed feeding improvement on the total and regional residual brain volume

Brain MRI at TEA was used to examine the vulnerable brain regions affected by early-life delayed feeding improvement. At TEA, 54 infants in the improvement group and 33 in the delayed improvement group underwent MRI assessments. Despite similar gestational age, gender, and postmenstrual age at MRI examinations between the two groups, the delayed improvement group had significantly smaller residual brain volumes involving intracranial volume, total brain volume, total tissue volume, brainstem, and cerebellum than the improvement group (Table 4).

**Table 4:** Total and regional residual brain volume between improvement and delayed improvement groups at term-equivalent age

	**Feeding improvement** ***N* = 54**	**Delayed feeding improvement** ***N* = 33**	*p*-value
Gestational age, mean ± SD	25.7 ± 1.9	25.7 ± 1.9	0.943
Gender, *n* (%)	29 (54)	22 (67)	0.268
PMA at MRI, weeks, mean ± SD	42.0 ± 3.8	43.7 ± 5.9	0.292
^#^ Residual brain volume, cm^3^, mean ± SD			
Intracranial volume (ICV)	12.3 ± 59.6	− 20.2 ± 66.0	0.020
Total brain volume (TBV)	10.8 ± 42.3	− 17.7 ± 48.2	0.026
Total tissue volume (TTV)	10.3 ± 40.3	− 16.9 ± 50.0	0.028
TTV minus brainstem/cerebellum	9.1 ± 37.1	− 14.8 ± 44.8	0.031
Brainstem	0.18 ± 0.72	− 0.29 ± 0.79	0.011
Cerebellum	0.05 ± 0.20	− 0.08 ± 0.30	0.010

*ICV* intracranial volume, brain grey matter tissue + white matter tissue + ventricles + extra-axial cerebrospinal fluid, *TBV* total brain volume, brain grey matter tissue + white matter tissue + ventricles, *TTV* total tissue volume, brain grey matter tissue + white matter tissue, *PMA* postmenstrual age, *MRI* magnetic resonance imaging

Student *t* test was used for continuous variables, chi-square test was used for categorical variables, and Mann–Whitney test was used for data that are not normally distributed

^#^Residual brain volume: $R_i=Y_i-\widehat{Y_i,}$ where ${R}_{i}$ is brain volume residual, ${Y}_{i}$ is brain volume, and ${\widehat Y}_i=b_0+b_1\times\mathrm{Male}+b_2\times\mathrm{PMA}+b_{\mathit2}\times\mathrm{PMA}^2$ where $b_i$’s are corresponding estimated coefficients by calculating from the linear regression model

### Alterations of metabolic pathways after delayed improvement in feeding progression

**Fig. 3:**
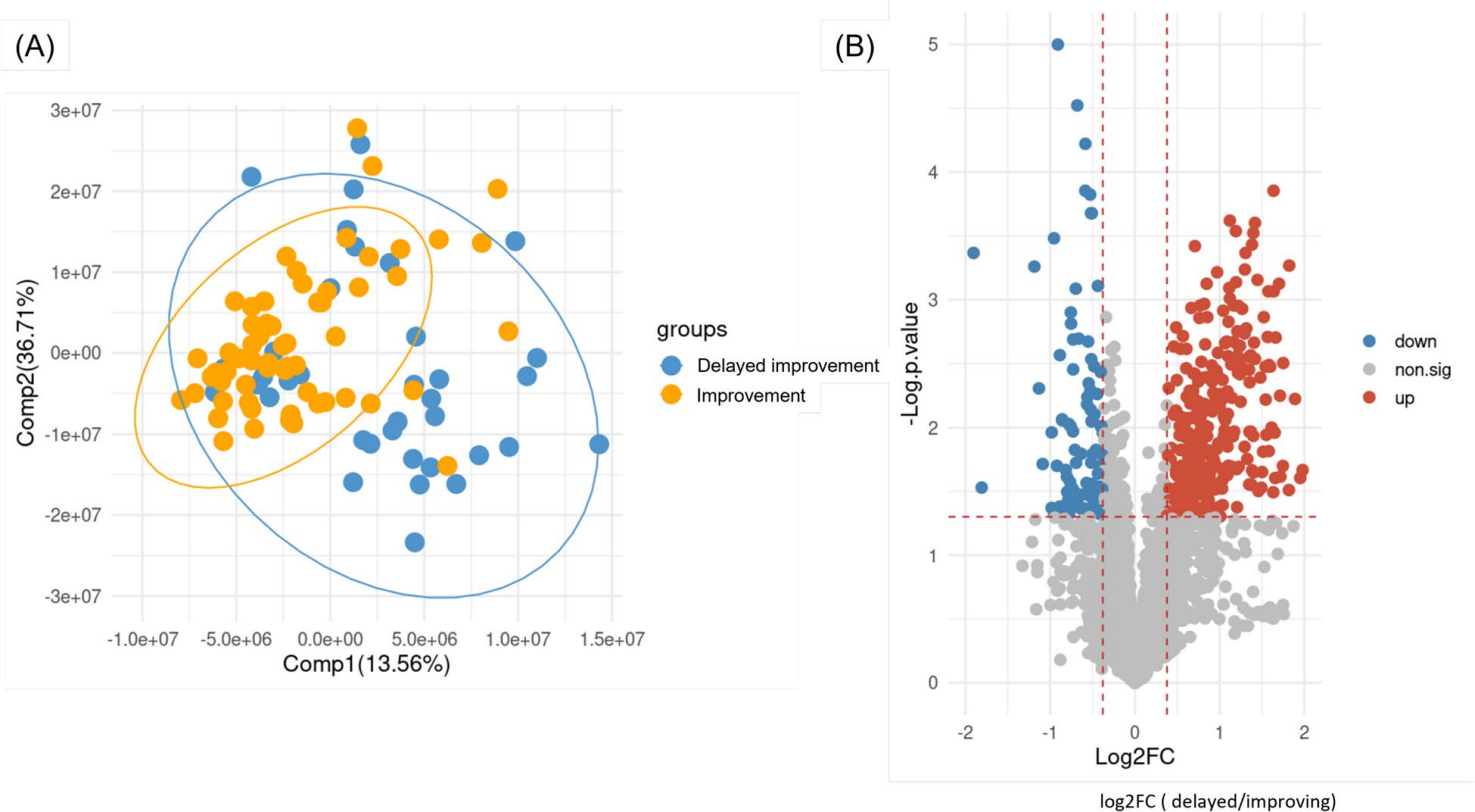
Plasma metabolomic profiles between two different feeding progression groups. **A** PLS-DA plot of the plasma samples from the delayed improvement and improvement groups. The improvement group (orange points) were more tightly clustered, indicating a more homogeneous distribution while the delayed improvement group (blue points) were more widely dispersed, indicating greater variability within this group. The ellipse outlines surrounding the points represented the 95% confidence intervals for each group. **B** Volcano plot compared the differentially expressed metabolites or differential abundance metabolites (DAMs) between the two groups. Each point on the plot represents an individual metabolite. The x-axis represents the log2 fold changes (log2FC) between the delayed improvement and the improvement group, while the *y*-axis represents the negative logarithm of the *p*-value [-Log(*p*-value)], indicating the statistical significance of the differences in metabolite levels. The vertical red dashed lines correspond to a fold change threshold of 1.3, while the horizontal red dashed line represents a *p*-value threshold of 0.05. The red points indicate metabolites that were significantly upregulated in the delayed improvement group, while the blue points indicate metabolites that were significantly upregulated in the improvement group. The grey points represent the metabolites with no significant differences between the two groups

The PLS-DA analysis of the plasma samples demonstrated that Components 1 (Comp1) and 2 (Comp2) collectively account for approximately 50.27% of the total variance, with Comp1 contributing 13.56% and Comp2 accounting for 36.71% (Fig. 3A). There were separations between the two different feeding progression groups. The distribution in the improvement group were more tightly clustered than that in the delayed improvement group. The improvement group also exhibited a smaller, more compact ellipse, reflecting homogeneity within group, whereas the delayed improvement group showed a larger, more scattered ellipse, indicating greater variability.

**Fig. 4:**
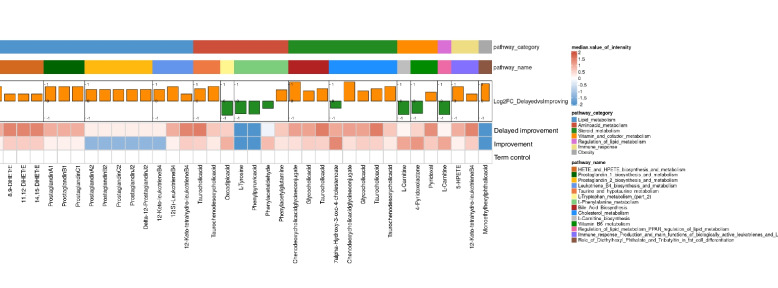
Visualization of the differences in metabolomics and pathways between the delayed improvement and improvement groups. The uppermost bar categorizes the metabolic pathways. The second bar lists the pathway names as identified by MetaCore. The third bar illustrates the log2 fold changes for each compound, comparing conditions between the delayed improvement group and the improvement group. At the bottom, the Heatmap represents the expression levels (intensity) of each metabolite in the delayed improvement and improvement groups, with the term-birth control serving as a baseline. Each column at the bottom represents a DAM-related compound. The Heatmap depicts the expression levels (intensity) of each DAM-related compound in the delayed improvement and improvement groups. To facilitate comparison between the delayed improvement and improvement groups, the intensity values for these groups were adjusted by subtracting the values from term-birth controls. Therefore, the color red indicates higher expression levels relative to the control, while the color blue indicates lower expression levels. The bar chart, located above the Heatmap, presents the log2 fold change (log2FC) between the delayed improvement and improvement groups for each DAM-related compound. Orange bars indicate higher expression in the delayed improvement group, while green bars indicate higher expression in the improvement group. The two horizontal lines at the top indicate the pathway names and pathway categories corresponding to each DAM-related compound. The legend on the right provides a color-coded key for the pathway categories and a scale for the median value of intensity

The volcano plot for the DAMs showed the delayed improvement group had a greater number of metabolites that were upregulated as compared to the improvement group (Fig. 3B). Figure 4 presents a comprehensive visualization of the DAM-related compounds between the two groups, with an additional reference to the 19 term-birth controls. The DAM-related compounds were primarily associated with the pathway catalogs in the metabolisms of lipid, amino acids and steroid, immune responses, and obesity. Notably, the Heatmap revealed that compared to the improvement group, the delayed improvement group had significantly elevated expression levels of compounds involving in the pathways of hydroxyeicosatetraenoic acid (HETE) and hydroperoxyeicosatetraenoic acid (HPETE), leukotriene B4, prostaglandin 1 and 2, bile acids, taurine, cholesterol and immune responses. The delayed improvement group also showed significantly lower expression levels of compounds involving L-tyrosine, phenylpyruvic acid and L-tryptophan metabolism, and L-carnitine biosynthesis (Additional File 1: Table S2**)**.

**Fig. 5:**
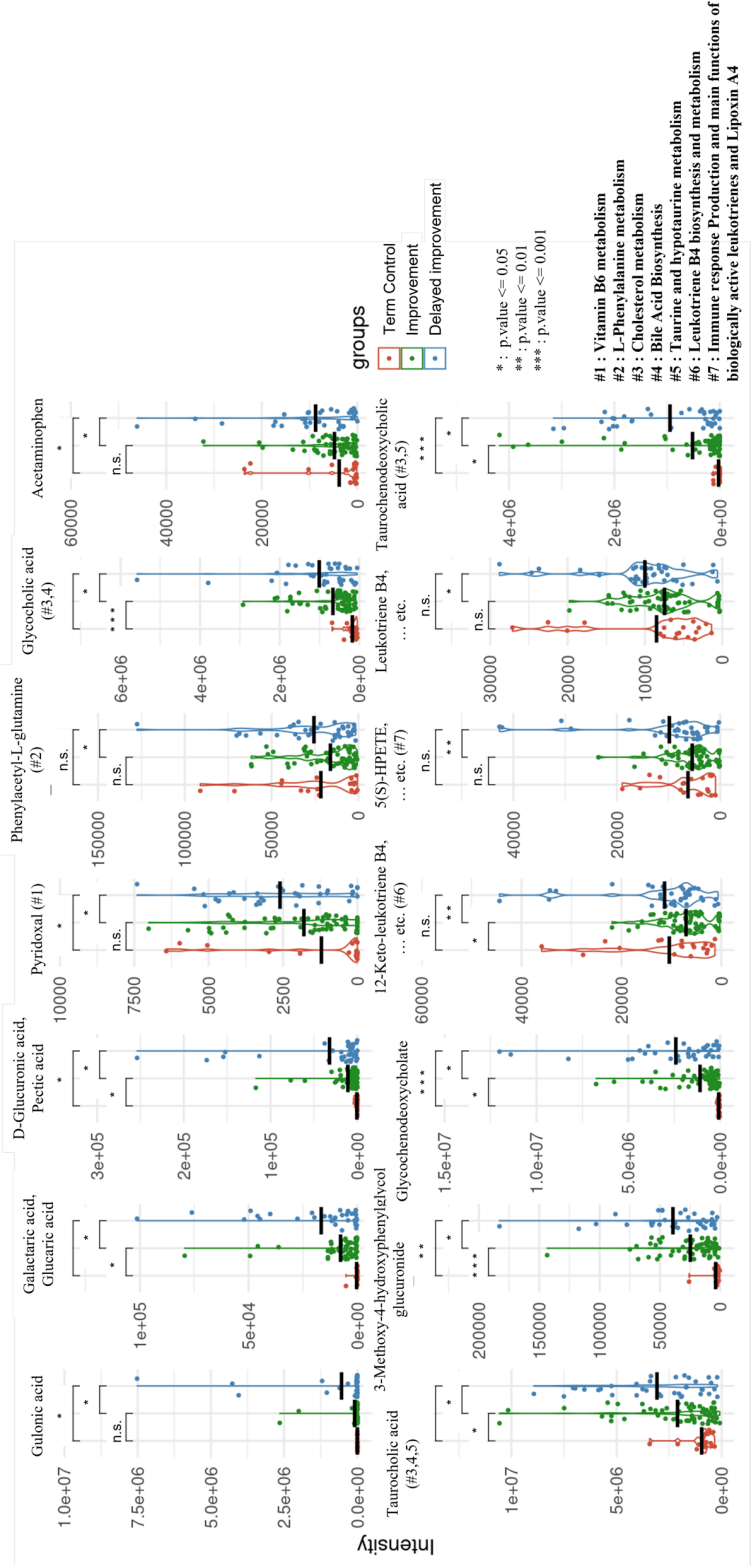
Distribution of DAM-related compounds (higher intensity). The DAM-related compounds that were of higher intensity in the delayed improvement group (blue dots) compared to those in the improvement group (green dots) and term-birth controls (solid red dots). For compounds predicted from the same metabolite, the plot highlights the compound with the highest evaluated score. Asterisk (^*^): significant differences between the groups; n.s.: no significant difference; black lines: the mean value of compounds in each group. #: involved pathways

**Fig. 6:**
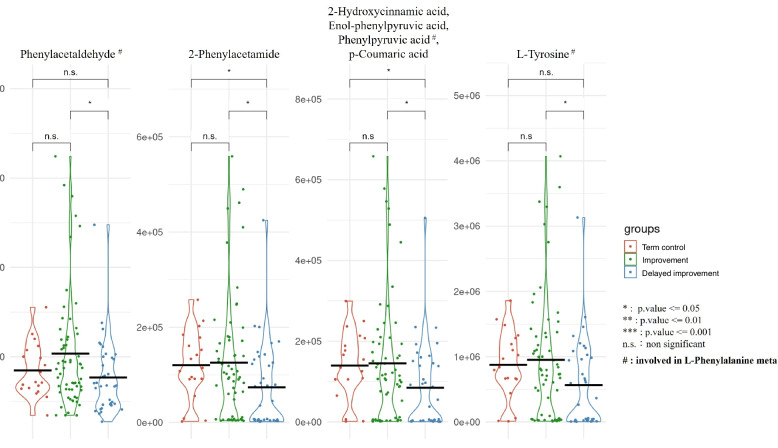
Distribution of DAM-related compounds (lower intensity). The DAM-related compounds that were of lower intensity in the delayed improvement group (blue dots) compared to those in the improvement group (green dots) and term-birth controls (solid red dots). For compounds predicted from the same metabolite, the plot highlights the compound with the highest evaluated score. Asterisk (*): significant differences between the groups; n.s.: no significant difference; black lines: the mean value of compounds in each group. #: involved pathways

Further analyses using the samples from term-birth neonates as baseline were undertaken to show the differences of DAM-related compound between the two different feeding progression groups. Compared to the improvement group, the delayed improvement group exhibited significantly higher intensity in the DAM-related compounds, such as gulonic acid, D-glucuronoic acid, galactaric acid, glycocholic acid, taurocholic acid, glucuronide, glycochenodeoxycholate, 12-keto-leukotriene B4, 5(s)-HPETE, and taurochenodeoxycholic acid. These compounds were involved mainly in the pathways of cholesterol metabolism, bile acid biosynthesis, Taurine and hypotaurine metabolism, leukotriene B4 biosynthesis and metabolism, and immune response production and main functions of biologically active leukotrienes and lipoxin A4 (Fig. 5). The delayed improvement group also had significantly lower levels of compounds, including phenylacetaldehyde, 2-phenylacetamide, phenylpyruvic acid, and L-tyrosine metabolism (Fig. 6). Notably, three of these four compounds were involved in the L-phenylalanine metabolism.

## Discussion

**Fig. 7:**
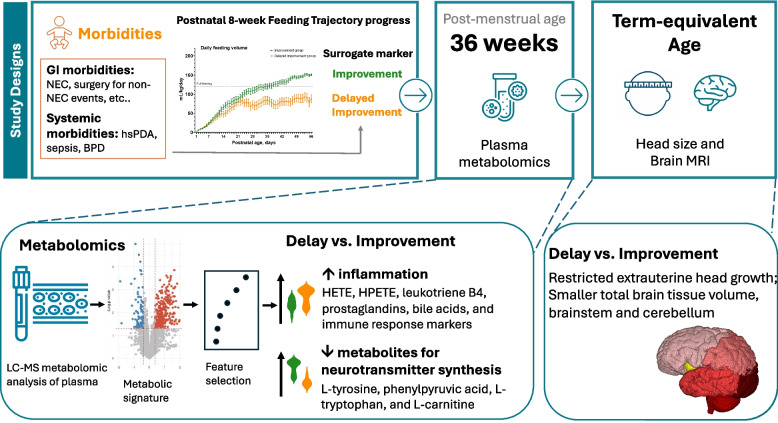
A graphic abstract of this study

Adequate nutritional supply is crucial during the critical brain growth period of extremely preterm infants in the NICU [63]. However, enteral feeding progression is often delayed due to immature gut function and related morbidities resulting in feeding intolerance, a common phenomenon seen in the NICU [64]. Previous research has indicated that delayed improvement in feeding progression is associated with an increased risk of neurodevelopmental impairment outcomes [23]. This study expands on prior findings [34] by demonstrating that early-life delayed feeding improvement, a surrogate marker of underlying morbidities such as NEC, non-NEC surgical abdominal events and BPD during admission, is significantly associated with selective EUGR in HC at TEA. This restriction correlates with reduced total brain tissue volumes, including the brainstem and cerebellum. To explore the underlying metabolic mechanisms, untargeted plasma metabolomics was utilized, revealing that compared to the infants with feeding improvement, the infants with delayed improvement in feeding progression exhibited elevated levels of HETE, HPETE, leukotriene B4 [65], prostaglandins [66], bile acids [46], and immune response markers, indicating a heightened inflammatory response. In contrast, they also showed significantly reduced levels of metabolites involved in neurotransmitter synthesis and energy metabolism, including L-tyrosine [67], phenylpyruvic acid, L-tryptophan metabolism [68], and L-carnitine biosynthesis [69]. These findings suggest that delayed feeding improvement in early-life, as a surrogate marker for the underlying gastrointestinal and systemic morbidities, may contribute to a proinflammatory state and metabolic dysregulation, which could mediate the relationship between poor feeding progression and restricted brain growth. This highlights the crucial role of the gut-brain axis in the critical care of extremely preterm infants in the NICU (Fig. 7).

Nutritional supply in preterm infants plays a critical role in promoting growth velocity, increasing HC, and supporting the maturation of cerebral white matter connective tracts [70, 71]. Head growth serves as a crucial clinical surrogate marker for brain growth, as the volumetric expansion of the developing brain is the primary driver of head growth [72]. A prior study established this link by comparing total cerebral volume, quantified from MRI scans, with occipital-frontal head circumference measurements, both obtained at term-equivalent age [73]. This study is the first to demonstrate that delayed feeding progression in the NICU is not only associated with selective EUGR in HC but also with widespread reductions in brain volume, including the brainstem and cerebellum.

Two studies have demonstrated an association between altered plasma metabolome and EUGR in BW among very preterm infants [36, 44]. One study reported a progressive decline in glycerophospholipids, sphingolipids, and other lipid levels as the severity of growth restriction increased [44]. Another study found that preterm infants with EUGR in BW, but without SGA at birth, exhibited distinct metabolomic profiles characterized by altered levels of sphinganine-1-phosphate and 2-oleoylglycerol [36]. However, both studies focused solely on EUGR in BW, without addressing its effects on HC. Additionally, the *z*-scores for both BW and HC at birth were already significantly lower in the EUGR group compared to the non-EUGR group in the study from Dudzik et al. [44]. Our feeding progression study further revealed that significant differences in HC between the two groups emerged by TEA, with the delayed improvement group exhibiting significantly lower HC *z*-scores and delta *z*-scores. Moreover, metabolomics analysis revealed a significant increase in metabolites associated with pro-inflammatory pathways, including HPETE, leukotriene B4, and bile acids. In contrast, metabolites involved in the biosynthesis of L-tyrosine, L-tryptophan, and L-carnitine were markedly reduced in the delayed improvement group.

Previous studies have shown that systemic increases in pro-inflammatory cytokines can activate microglia, reduce neurogenesis, and impair myelination, ultimately leading to altered brain development and poorer cognitive outcomes in preterm infants [74–76]. Cysteinyl leukotrienes (CysLTs), synthesized from arachidonic acid via the 5-lipoxygenase metabolic pathway, are potent pro-inflammatory mediators that have been implicated in white matter injury, NEC and BPD [77–79]. Our study found that the delayed improvement group had significantly higher rates of severe NEC and moderate to severe BPD compared to the improvement group. Further metabolomic analysis identified elevated levels of pro-inflammatory metabolites, including leukotrienes and 15-HPETE, following delayed feeding improvement. The prostanoid, leukotriene, and HPETEs biochemical pathways may include specific enzymes of the cyclooxygenases and lipoxygenases families [80]. These systemic inflammatory molecules may be strongly associated with impaired head growth and reduced brain volume at TEA in infants with delayed feeding improvement. However, whether this pro-inflammatory state arises primarily from nutritional status [81] or is linked to gastrointestinal morbidities remains unclear and requires further investigation.

We found that the delayed improvement group exhibited significantly higher plasma levels of bile acids, including glycocholic acid and taurocholic acid, compared to both the improvement group and the term-birth control group. These findings align with previous studies reporting elevated bile acid metabolites in the plasma of very preterm infants with severe EUGR in BW, and of preterm infants with brain injury [44, 46]. Bile acid metabolism plays a crucial role in several neurological disorders [82]. Increased serum levels of glycocholic acid and taurocholic acid have been observed in patients with liver cirrhosis and hepatic encephalopathy [83]. In murine models of hepatic encephalopathy due to acute liver failure, elevated bile acid levels were associated with neurological decline, while suppression of bile acid signaling pathways in the frontal cortex partially mitigated these complications [84]. Mechanistically, taurocholic acid may contribute directly to neuroinflammation via sphingosine-1-phosphate receptor 2 signaling [85]. Moreover, bile acid metabolism is closely linked to gut microbiota function, and these metabolic differences may reflect variations in intestinal colonization [86]. Emerging evidence suggests that bile acids play a key role in promoting pro-inflammatory responses, gut bacterial overgrowth, and intestinal inflammation [87, 88]. In our study, the delayed improvement group had a significantly higher incidence of NEC of Bell stage II or higher and non-NEC surgical abdomen events compared to the improvement group. These findings suggest alterations in gut microbiota, as a key mediator with neuroinflammatory features through production of the bioactive metabolites, may contribute to impaired brain growth following delayed improvement in feeding progression.

Nutrient supply to preterm infants has been associated with blood amino acid concentrations and growth patterns [70]. Previous studies have revealed a link between EUGR and disrupted amino acid metabolism—particularly involving tyrosine, tryptophan, and phenylalanine—as well as impaired carnitine biosynthesis in preterm infants [44]. The phenylalanine metabolic pathway, which includes tyrosine, plays a crucial role in producing neurotransmitters such as dopamine, epinephrine, and norepinephrine [89]. In this study, the delayed improvement group exhibited significantly lower levels of phenylacetaldehyde, 2-phenylacetamide, and tyrosine, indicating disruptions in this metabolic pathway. Similarly, tryptophan, a precursor of serotonin, is essential for the microbiota-gut-brain axis and the development of both the central and enteric nervous systems [68]. Although carnitine is not a neurotransmitter precursor, it is vital for mitochondrial function, as it facilitates the transport of long-chain fatty acids into the mitochondrial matrix. Prior research has also highlighted the relationship between mitochondrial function and serotonin, with potential implications for neurodegenerative diseases [90]. Extremely preterm infants are at risk of developing carnitine deficiency due to limited tissue stores, immature endogenous synthesis, and insufficient dietary intake. This deficiency is further exacerbated by their increased carnitine requirements during rapid growth [69]. However, limited research has explored the relationship between carnitine levels and head growth in preterm infants. In this study, we found that delayed feeding improvement was associated with significantly lower plasma levels of tyrosine, L-phenylalanine-related metabolites, and L-carnitine by postmenstrual age 36 weeks, as well as reduced brain growth at TEA. These findings suggest a potential link between feeding progression, these amino acids and immature brain development.

This study has several limitations. First, its observational design limits our ability to establish a causal relationship between delayed feeding progression and the observed metabolic and neurodevelopmental outcomes. Second, the interpretation of our metabolomic findings is complex. The delayed improvement group exhibited higher rates of gut and BPD morbidities. These factors, as well as some unmeasured parameters, such as antibiotics exposure, are known to influence the gut microbiome and metabolic profiles. Therefore, delayed feeding progression should be interpreted not as an isolated factor, but as a clinical surrogate marker reflecting this multifaceted state of vulnerability in a certain group of preterm infants, which collectively contributes to the observed metabolomic signatures. Third, our use of a term-infant cohort as a physiological reference is constrained by the significant differences in postnatal age and the developmental stage of the gut microbiome. Finally, this study did not include direct analysis of the gut microbiota. Longitudinal studies that integrate clinical data with serial gut microbiota and metabolomic analyses are essential to fully elucidate the mechanisms of the gut-brain axis in this vulnerable population.

## Conclusions

This study suggests that early-life delayed improvement in feeding progression is associated with disruptions in characteristic metabolomic signatures and reduced head growth in extremely preterm infants. The gut-brain axis findings underscore the importance of closely monitoring preterm infants with delayed feeding progression and exploring potential early metabolomic interventions to support optimal brain growth.

## Supplementary Information

Additional File 1: Table S1-S2 and Fig S1. Table S1. Definitions of neonatal morbidities. Table S2. Pathway enrichment analysis of differences in DAM-related compounds between the delayed improvement and improvement groups ranked by False Discovery Rate (FDR) using MetaCore. Fig S1. Workflow for plasma metabolomics analysis.

## Data Availability

All data are included in the manuscript and supporting information. The datasets used and/or analyzed during the current study are available from the corresponding author on reasonable request.

## Abbreviations

BPD: Bronchopulmonary dysplasia
BW: Bodyweight
DAMs: Differential abundance metabolites
EUGR: Extrauterine growth restriction
HC: Head circumference
HETE: Hydroxyeicosatetraenoic acid
HPETE: Hydroperoxyeicosatetraenoic acid
hsPDA: Hemodynamically significant patent ductus arteriosus
LC-MS: Liquid chromatography-mass spectrometry
NEC: Necrotizing enterocolitis
NICU: Neonatal intensive care unit
PLS-DA: Partial Least Squares Discriminant Analysis
QToF: Quadrupole-time-of-flight
ROP: Retinopathy of prematurity
SGA: Small for gestational age
TEA: Term equivalent age
