# Supplementary material for: Proinflammatory state and metabolic dysregulation linking delayed feeding progression to extrauterine restricted head growth in extremely preterm infants
Source: BMC Med. 2025 Dec 29;23:701. doi: 10.1186/s12916-025-04525-w (PMC12750882; doi:10.1186/s12916-025-04525-w)
Supplement: Supplementary file 1 — Additional File 1: Table S1-S2 and Fig S1. Table S1. Definitions of neonatal morbidities. Table S2. Pathway enrichment analysis of differences in DAM-related compounds between the delayed improvement and improvement groups ranked by False Discovery Rate (FDR) using MetaCore. Fig S1. Workflow for plasma metabolomics analysis. [file 12916_2025_4525_MOESM1_ESM.docx]

Additional File 1 Table S1. Definitions of neonatal morbidities

| **Neonatal morbidities** | **Definitions** |
| --- | --- |
| Small for gestational age [1] | Defined as birth body weight below the 10^th^ percentile for sex and gestational age according to the Fenton growth chart. |
| Preeclampsia [2] | Pregnancy-induced hypertension (arterial blood pressure >140/90 mm Hg) during the second half of gestation. |
| Severe brain injury | Defined as intraventricular hemorrhage grade III or any grade of intraventricular hemorrhage plus periventricular hemorrhage, or cystic periventricular leukomalacia.  Grade of intraventricular hemorrhage was based on the Papile classification system [3]. Cystic periventricular leukomalacia was defined as periventricular echolucent cystic lesions or loss of periventricular white matter with ventriculomegaly and irregular margins by ultrasonography. Defined as periventricular echolucent cystic lesions or loss of periventricular white matter with ventriculomegaly and irregular margins by ultrasonography [4]. |
| Hemodynamically significant patent  ductus arteriosus [5] | Based by clinical indices (a cardiovascular distress score ≥3) and confirmed by echocardiographic investigation (LA/Ao≥1.3~1.4) and requiring surgery or medication interventions. |
| Bronchopulmonary dysplasia [6] | Defined by Jensen 2019 definition, according to the types of respiratory support (grade 1: nasal cannula ≤ 2 L/min; grade 2, Nasal cannula >2 L/min or nCPAP or nasal IPPV; and grade 3, invasive ventilator) assessed at 36 weeks’ PMA. Moderate/severe BPD: BPD grade ≥ II. |
| Sepsis [7] | Defined according to the presence of a positive blood culture. |
| Necrotizing enterocolitis of Bell stage II or higher [8] | Defined based on modified Bell staging classification. Necrotizing enterocolitis ≥ stage 2 was included for the current analysis. |
| Severe retinopathy of prematurity [9] | Defined as stage 2 plus or worse, or requiring retinal therapy |

**Additional File 1 Table S2.** Pathway enrichment analysis of differences in DAM-related compounds between the delayed improvement and improvement groups ranked by False Discovery Rate (FDR) using MetaCore.

| **Pathway_Map (MetaCore)** | **FDR** | DAM-related compounds |
| --- | --- | --- |
| HETE and HPETE biosynthesis and metabolism | 1.90E-06 | 12(S)-HPETE;  15(S)-HPETE;  8,9-DiHETrE;  11,12-DiHETrE;  14,15-DiHETrE |
| L-Phenylalanine metabolism | 3.05E-04 | L-Tyrosine;  Phenylpyruvicacid;  Phenylacetaldehyde;  Phenylacetylglutamine; |
| Leukotriene B4 biosynthesis and metabolism | 3.05E-04 | 12-Keto-leukotrieneB4;  12(S)-LeukotrieneB4;  12-Keto-tetrahydro-leukotrieneB4 |
| Prostaglandin 2 biosynthesis and metabolism | 3.05E-04 | ProstaglandinA2;  ProstaglandinB2;  ProstaglandinC2;  ProstaglandinJ2;  Delta-12-ProstaglandinJ2; |
| Cholesterol metabolism | 3.05E-04 | 7alpha-Hydroxy-3-oxo-4-cholestenoate;  Chenodeoxycholicacidglycineconjugate;  Glycocholicacid;  Taurocholicacid;  Taurochenodesoxycholicacid |
| L-Carnitine biosynthesis | 2.54E-03 | L-Carnitine |
| Regulation of lipid metabolism_PPAR regulation of lipid metabolism | 8.30E-03 | L-Carnitine |
| Immune response_Production and main functions of biologically active leukotrienes and Lipoxin A4 | 8.71E-03 | 5-HPETE;  12-Keto-tetrahydro-leukotrieneB4 |
| Prostaglandin 1 biosynthesis and metabolism | 1.13E-02 | ProstaglandinA1;  ProstaglandinB1;  ProstaglandinC1 |
| Taurine and hypotaurine metabolism | 2.39E-02 | Taurocholicacid;  Taurochenodesoxycholicacid |
| Role of Diethylhexyl Phthalate and Tributyltin in fat cell differentiation | 3.50E-02 | Monoethylhexylphthalicacid |
| Bile Acid Biosynthesis | 3.52E-02 | Chenodeoxycholicacidglycineconjugate;  Glycocholicacid;  Taurocholicacid |
| L-Tryptophan metabolism (part 2) | 3.55E-02 | Oxoadipicacid |
| Vitamin B6 metabolism | 4.02E-02 | 4-Pyridoxolactone;  Pyridoxal |


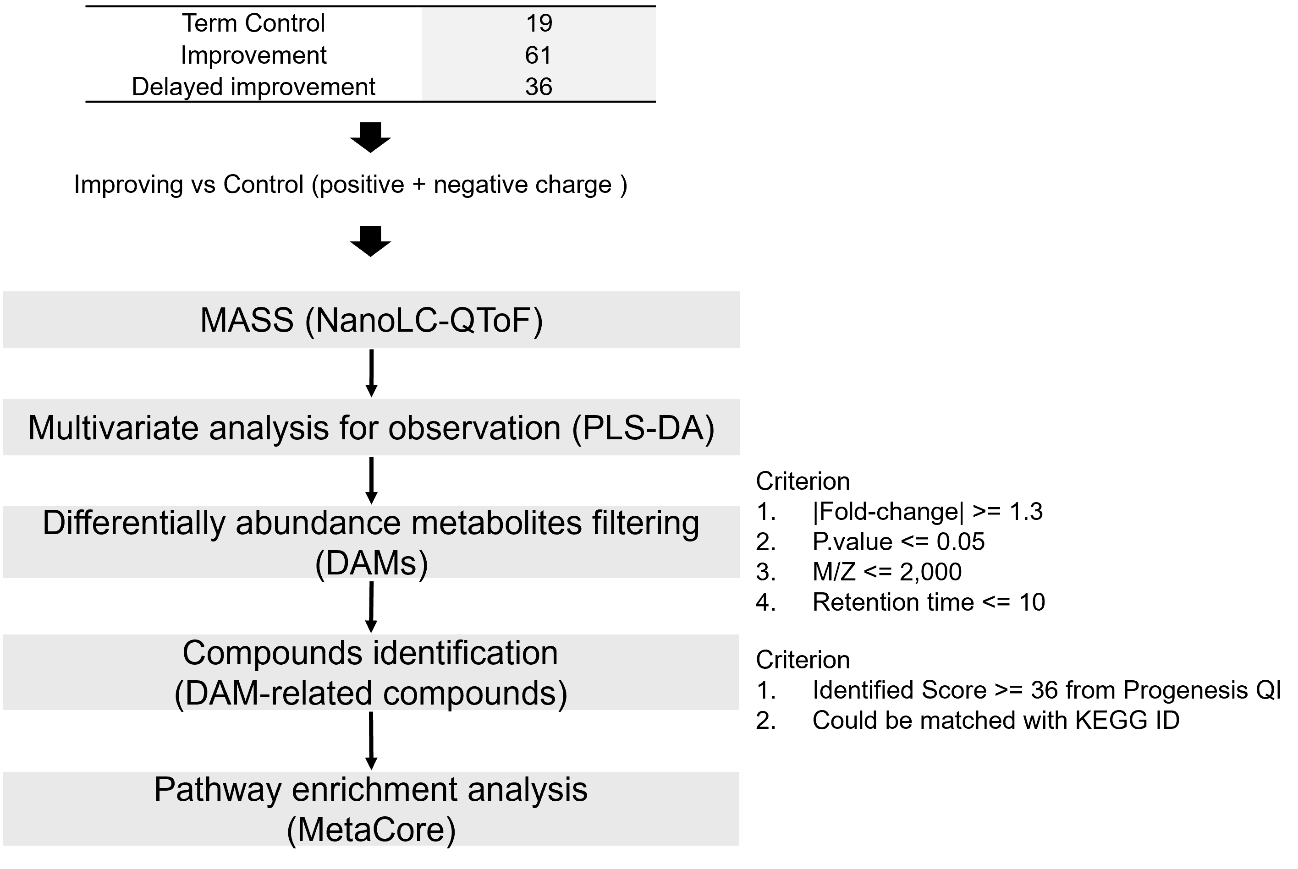
**Additional File 1 Fig S1.** Workflow for plasma metabolomics analysis.

**References**

1. Fenton TR, Kim JH: **A systematic review and meta-analysis to revise the Fenton growth chart for preterm infants**. *BMC Pediatr* 2013, **13**:59.

2. **Gestational Hypertension and Preeclampsia: ACOG Practice Bulletin Summary, Number 222**. *Obstet Gynecol* 2020, **135**(6):1492-1495.

3. Valdez Sandoval P, Hernandez Rosales P, Quinones Hernandez DG, Chavana Naranjo EA, Garcia Navarro V: **Intraventricular hemorrhage and posthemorrhagic hydrocephalus in preterm infants: diagnosis, classification, and treatment options**. *Childs Nerv Syst* 2019, **35**(6):917-927.

4. Deng W, Pleasure J, Pleasure D: **Progress in periventricular leukomalacia**. *Arch Neurol* 2008, **65**(10):1291-1295.

5. Gillam-Krakauer M, Reese J: **Diagnosis and Management of Patent Ductus Arteriosus**. *Neoreviews* 2018, **19**(7):e394-e402.

6. Jensen EA, Dysart K, Gantz MG, McDonald S, Bamat NA, Keszler M, Kirpalani H, Laughon MM, Poindexter BB, Duncan AF *et al*: **The Diagnosis of Bronchopulmonary Dysplasia in Very Preterm Infants. An Evidence-based Approach**. *Am J Respir Crit Care Med* 2019, **200**(6):751-759.

7. Wynn JL: **Defining neonatal sepsis**. *Curr Opin Pediatr* 2016, **28**(2):135-140.

8. Neu J: **Necrotizing enterocolitis: the search for a unifying pathogenic theory leading to prevention**. *Pediatr Clin North Am* 1996, **43**(2):409-432.

9. **Screening examination of premature infants for retinopathy of prematurity**. *Pediatrics* 2001, **108**(3):809-811.
